# Study on the TCM Syndromes Evolution and Chinese Herbal Characteristics of Type 2 Diabetes Patients with Different Courses of Disease in TCM “Heat Stage”: A Real-World Study

**DOI:** 10.1155/2021/1282957

**Published:** 2021-06-16

**Authors:** Ying Xing, Min Pi, Runshun Zhang, Tiancai Wen

**Affiliations:** ^1^Institute of Basic Research in Clinical Medicine, China Academy of Chinese Medical Sciences, Beijing, China; ^2^Shenzhen Traditional Chinese Medicine Hospital, Guangdong, China; ^3^Guang'anmen Hospital, China Academy of Chinese Medical Sciences, Beijing, China; ^4^Traditional Chinese Medicine Data Center, China Academy of Chinese Medical Sciences, Beijing, China

## Abstract

**Objective:**

The purpose of this study is to analyze and summarize the syndrome distribution, syndrome evolution, and Chinese herb medicine characteristics of T2D in heat stage.

**Method:**

In this study, 228 heat-stage T2D patients were divided into three groups based on the course of disease. Group 1 (the course of disease ≤5 years) included 118 patients. Group 2 (5< the course of disease ≤10 years) had 73 patients. Group 3 (the course of disease >10 years) consisted of 37 patients. The main methods used in our study were complex network community partitioning algorithms and Sankey diagram visualization, based on the clinical electronic medical record data we collected.

**Result:**

In the three groups, the nodes with the highest node degree are all “heat syndrome.” Edge weight between “heat” and “dampness,” “qi stagnation,” “phlegm,” “liver,” and “stomach” is the largest. During the whole course of treatment, 60.17%, 63.01%, and 62.16% of the patients' syndromes in groups 1, 2, and 3, respectively, were ascribed to the heat stage all the time. The patients' syndromes in groups 1 and 2 easily transformed to the syndrome of deficiency of both qi and yin of the spleen and stomach. In group 3, 27% of the patients' syndromes were easily transformed into kidney yin deficiency and qi deficiency and blood stasis syndrome. The largest Chinese herb communities of the patients whose syndromes did not change after treatment in the three groups were all heat-clearing drugs. The proportion of blood-activating drugs in patients with syndrome changes increased significantly after treatment.

**Conclusion:**

(1) The basic syndrome of T2D patients in the heat stage is liver-stomach heat syndrome. (2) T2D patients in the heat stage tend to deteriorate towards the direction of qi and yin deficiency syndrome. However, the longer the course of the disease is, the more likely it is to deteriorate to the direction of kidney yin deficiency syndrome and blood stasis syndrome. (3) Drugs that can help T2D patients in the heat stage to maintain their condition stably are heat-clearing drugs represented by *Coptis chinensis*, which usually need to be combined with warming interior drugs such as Zingiberis Rhizoma and Pinelliae Rhizoma.

## 1. Introduction

Type 2 diabetes (T2D), namely, “xiaoke” of TCM, is a chronic and complex disease with diverse etiologies, long course, and different stages. In TCM, we concluded that diabetes had four different pathological stages based on clinical symptoms and manifestations, namely, stagnation stage, heat stage, deficiency stage, and injuring stage [1]. The heat stage is equivalent to the heat stage-to-mid period of diabetes and indicates the attack of disease [2]. Therefore, the heat stage is an extremely critical stage in the development of T2D. The study in [3] had shown that T2D patients in the heat stage are mostly obese, and the main syndrome is excess heat syndrome, but the specific type and the evolution direction of heat syndrome had not been explained clearly at present.

The course of disease is an important factor affecting the adverse control of blood glucose and chronic complications in T2D [4]. Related studies have revealed that patients with a course of more than 5 years have more than twice as much unsatisfactory blood glucose control as other patients [5], and the incidence of T2D retinopathy, nephropathy, and neuropathy will increase with the course of diabetes [6]. Considering the irreversibility of the increase in the course of the disease, at the key treatment point of the heat stage, if we can make clear the evolution direction of the syndrome of patients with different courses of disease and use effective Chinese herb medicine to treat them pertinently, then it will be of great help to prevent the aggravation of the disease or even to reverse the current poor state of the disease. But now we have not found related reports.

Clinical epidemiological investigation or clinical trial research had always been the golden standard for studying the evolution of syndrome. But, with the popularity of real-world study concepts, knowledge captured during routine clinical pathways in Electronic Medical Records (EMRs) has ushered a new way of syndromes evolution study that can provide evidence for medical decision-making beyond that from formal clinical studies. However, the current research [7–9] on syndrome evolution based on EMRs has an obvious shortcoming. Their method is usually to extract the distribution changes of syndrome frequency at different time points to describe the changes of syndrome, which will entirely isolate the syndrome at different time points and cannot completely describe the continuous evolution pattern of syndrome in the time dimension. In recent years, there are some new methods to study the evolution of syndrome, such as transition probability matrix [10], nonlinear mixed effect model [11], hidden structure model [12], and potential category analysis [13], but they are still rarely used in T2DM. Based on the above situation, this study mainly uses complex network community discovery algorithm and Sankey diagram to find out the syndrome evolution and Chinese herb medicine characteristics of T2D patients with different courses of disease in TCM heat stage, so as to provide reference for clinic.

## 2. Materials and Methods

### 2.1. Data Source and Data Screening Criteria

The real-world data used in this study are the EMRs of 590 T2D patients, which are obtained from the clinical data warehouse of the Traditional Chinese Medicine Data Center of the China Academy of Chinese Medical Sciences. We extracted the information we need from the EMRs, including the patient's unique code, treatment date, syndrome diagnosis, and Chinese herb medicine prescription and dosage. Since we need to include T2D patients in the heat stage, we refer to the definition of T2D heat stage in *international traditional Chinese medicine guideline for diagnostic and treatment principles of diabetes* [2], an international standard for T2D TCM treatment. When the patient's initial visit symptoms meet the criteria for the heat stage, then the patient is considered to be in the T2D heat stage. In addition, we have strict restrictions on the patient's total disease duration and the date interval between the adjacent prescriptions: the total number of visits ≥3 times. The total duration of treatment ≥ 30 days. The time span of adjacent prescriptions is between 7 and 30 ± 5 days. Our goal is to prevent certain special circumstances from affecting the accuracy of the results, such as too few visits (such as only one treatment) or too long interval between two treatments (such as a year from the last treatment).

### 2.2. Patient Grouping and Syndrome Division

We selected 228 patients who met the screening criteria from 590 patients. These patients were divided into three groups: Group 1 (the course of disease ≤5 years) included 118 patients. Group 2 (5< the course of disease ≤10 years) had 73 patients. Group 3 (the course of disease >10 years) consisted of 37 patients. In the real TCM clinical work scene, doctors' writing of TCM syndromes of patients is often not standard enough, such as the same syndrome but different ways of writing, which cause many restrictions to our research. Therefore, we disassembled all the syndromes into a combination of disease location syndrome elements and disease nature syndrome elements. The problem of syndrome heterogeneity is well solved by this method, which makes the clinical syndrome differentiation results more accurate and reliable. We refer to the *Syndrome Element Dialectics* [14] to split the syndrome.

### 2.3. Data Analysis

Our research mainly uses two kinds of data mining methods: complex network community partition algorithm and Sankey diagram. Here are several important concepts [15] that need our attention in complex networks: a node in a complex network is an entity. Edge is the relationship between nodes in a complex network, namely, the relationship between different entities in a complex system. Node degree is the number of edges connected to a node. Edge weight indicates the closeness of the relationship between the two nodes.

First of all, we first establish the undirected weighted complex network of T2DM syndrome and Chinese herb medicine. In this study, the node represents the syndrome element or Chinese herb medicine, and the edge signifies a pair of syndrome elements or the compatibility of Chinese herb medicine:

(1) $$\begin{array}{c} \deg\left(v_{i}\right)=w_{i}\sum_{i,j\in G}e\left(i,j\right). \end{array}$$

In formula (1), *G* is a complex network, and *v*_*i*_ represents node *I* in *G*. *e* (*i*, *j*) indicates whether there is an edge between nodes *I* and *j*; if it exists, the value is 1; otherwise it is 0. *w*_*i*_ represents the weight of node *I*, and the value of *w*_*i*_ in syndrome network is 1. In the traditional Chinese medicine network, *w*_*i*_ is expressed as the average dose of Chinese herb medicine.

Based on undirected weighted complex network, we use the FastUnfolding algorithm for community division. In complex networks, some nodes are closely connected and some are sparse. Then the tightly connected parts of the nodes can be regarded as a community, while the connections between communities are relatively sparse [16]. The FastUnfolding algorithm takes modularity as an index to divide closely connected nodes into a community [17]. In our study, the purpose of this algorithm is to find the clustering rule of syndrome and Chinese herb medicine in the heat-stage T2D population. Due to the large complex network caused by many kinds of Chinese herb medicine, we screened the nodes with a node degree ≥50 to find more core Chinese herb medicine. The establishment of complex networks and communities division all rely on Gephi 0.9.2.

**Figure 1:**
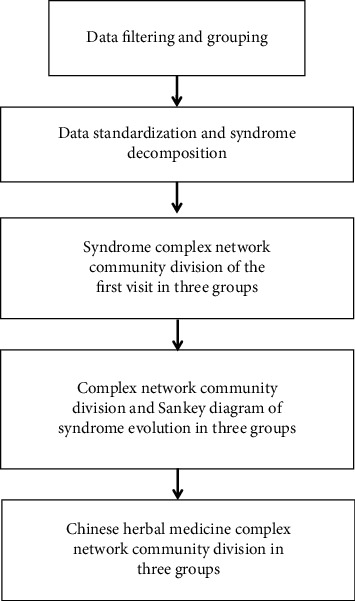
Data analysis flow chart.

Secondly, according to the statistical results of the outpatient visit distribution, we take the representative time point as the observation end point and draw the Sankey diagram of syndrome evolution. Sankey diagram, also known as heat balance diagram or energy flow chart, is composed of edges, flow rates, and nodes. Nodes represent different categories to divide different energy flow stages or zones. Edges connect nodes of different stages or zones, representing the flow of energy or data [18]. In this study, Sankey diagram was used to show the evolution of heat stage syndrome of T2DM. The node is the outpatient visit, the edge is the syndrome, and the width of the edge indicates the number of people who have syndrome transformation.  Figure 1 clearly reflects the data analysis process.

## 3. Results

### 3.1. Characteristics of the Participants

228 patients were enrolled in the study, with a total of 1435 outpatient visits. Most of patients in the three groups were male, and there was no statistical difference in gender composition among the three groups (*P*=0.864). Most of the patients in group 1 were 18∼40 years old and 40∼60 years old (*n* = 95, 41.67%). Most of the patients in group 2 were 40∼60 years old and over 60 years of age (*n* = 67, 29.39%). All the patients in group 3 were 40∼60 years old and over 60 years of age (*n* = 37, 16.23%), and no one was 18∼40 years old. The difference in age composition among the three groups was statistically significant (*P* < 0.001). The total number of overweight and obese patients in the three groups was 109 (71.24%). There was no statistically significant difference in BMI values among the three groups (*P*=0.383) (Table 1).

**Figure 2:**
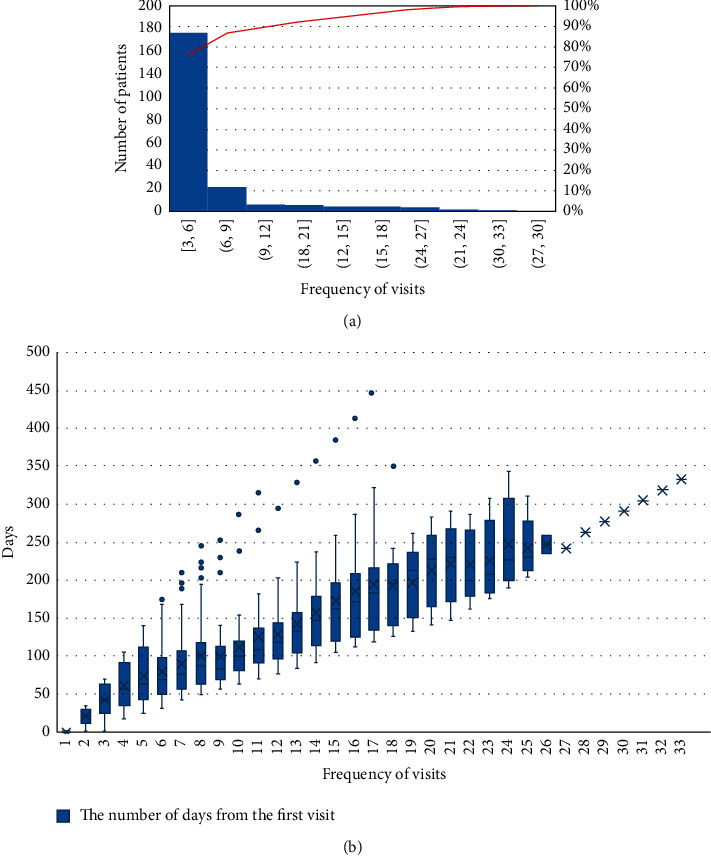
The distribution of visits and the time interval between hospital visits of T2D patients. (a) Cumulative distribution table of outpatient visits. (b) Boxplot of the number of days from the first visit.

The number of visits of most patients ranged from 3 to 5 (*n* = 174, 76.32%), and the average number of visits was 6.29 (Figure 2(a)). The number of days from the first visit of all patients remained stable before the 13th visit but then began to fluctuate sharply (Figure 2(b)).

### 3.2. Syndromes Distribution of T2D Patients in Heat Stage with Different Courses

**Figure 3:**
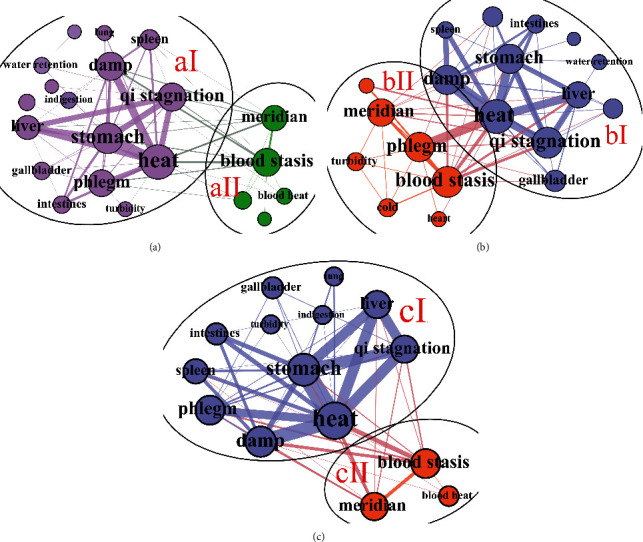
Syndrome complex network community division of T2D patients with different courses in the heat stage. (a) Syndrome complex network community division of group 1. (b) Syndrome complex network community division of group 2. (c) Syndrome complex network community division of group 3.

By analyzing the results of syndrome elements complex network community division of patients with different disease courses at the first visit, we found that the core syndrome combinations of patients in the three groups were highly similar. In the three groups, the largest syndrome communities (syndrome communities aI, bI, and cI) accounted for 56.52%, 57.14%, and 69.57%, respectively (Figures 3(a)–3(c)). Among the syndrome communities aI, bI, and cI, the syndrome nodes with the highest node degree are all “heat” (Figures 3(a)–3(c)). In addition, edge weight between “heat” and “dampness,” “qi stagnation,” “phlegm,” “liver,” and “stomach” is the largest (Figures 3(a)–3(c)). Syndrome communities aII, bII, and cII were the second largest syndrome communities in the three groups, accounting for 21.74%, 33.33%, and 21.74%, respectively, with blood stasis syndrome and meridians as the core nodes (Figures 3(a)–3(c)).

### 3.3. Syndromes Evolution of T2D Patients in Heat Stage

**Figure 4:**
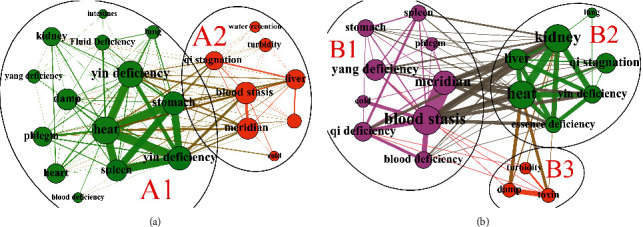
Complex network community division of syndrome evolution in heat stage. (a) Class A syndrome. (b) Class B syndrome.

The syndromes of T2D patients in the heat stage mainly evolved to two types of syndromes. The first is class A syndrome: the largest syndrome community A1 included 85.71% of syndrome element nodes, with heat, yin deficiency, qi deficiency syndrome and spleen, and stomach having the largest node degree and edge weight (Figure 4(a)). The second is class B syndrome: the largest syndrome community B1 accounted for 48.28%, and the node degree and edge weighting degree of qi deficiency, blood deficiency, blood stasis, yang deficiency, and meridians were the largest. The proportion of syndrome community B2 was 34.48%, and the node degree and edge weighting degree of heat, yin deficiency, essence deficiency, liver, and kidney were the highest (Figure 4(b)).

Since most of the T2D patients in this study were seen for follow-up visits within 6 times (Figure 2(a)), we intercepted the syndrome data of all patients from 1 to 6 visits to draw a Sankey diagram, and less than 6 times we regarded it as a loss of follow-up.

**Figure 5:**
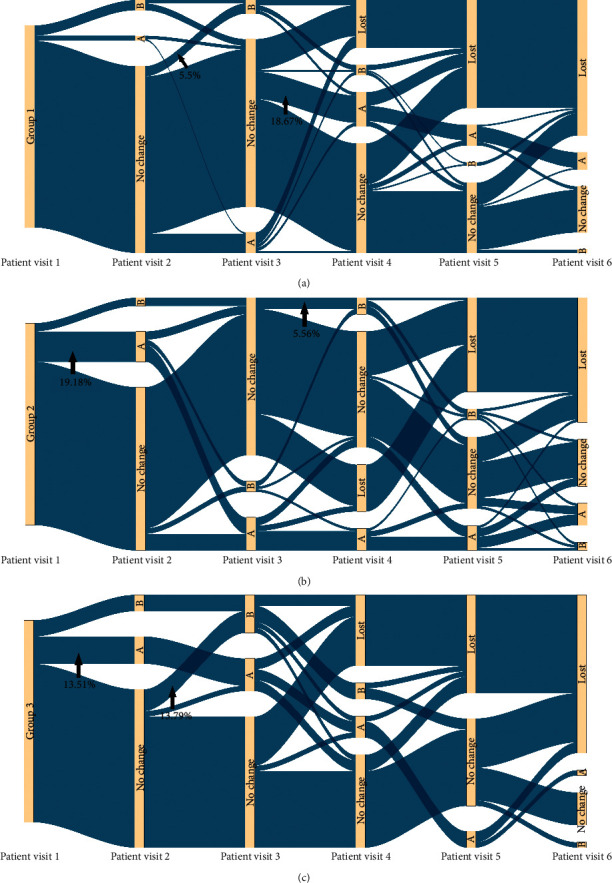
Sankey diagram of syndrome evolution of T2D patients in heat stage. (1) A: class A syndrome; (2) B: class A syndrome; (3) Lost: loss to follow-up patients; (4) No change: patients' syndrome had no change. (a) Syndrome evolution Sankey diagram of group 1. (b) Syndrome evolution Sankey diagram of group 2. (c) Syndrome evolution Sankey diagram of group 3.

For patients in group 1, the syndrome conversion rate reached the highest rate of 20% at the fourth consultation point (average of 61 days). It is important to note that 60.17% of the patients' syndromes always belong to the heat stage during treatment. Similarly, the highest conversion rate to class A syndrome was 18.67% at the fourth consultation (average of 61 days). However, the highest conversion rate of patients to class B syndrome was only 5.5%, which occurred at the third consultation (an average of 42.4 days) (Figure 5(a)).

For patients in group 2, the syndrome conversion rate was the highest (19.18%) at the second time point (average of 21 days). More specifically, during the whole course of treatment, 63.01% of the patients' syndromes were always ascribed to the heat stage. The conversion rate of patients to class A syndrome was the highest (15.07%) at the second consultation time point (average 21 days) and the highest to class B syndrome at the fourth consultation time point (average of 61 days) (5.56%) (Figure 5(b)).

Patients with a course of more than 10 years had the highest syndrome conversion rate of 21.62% at the second consultation point (average of 21 days). At the second consultation (average of 21 days), the conversion rate to class A syndrome was the highest (13.51%). At the third consultation point (average of 42.4 days), the conversion rate to class B syndrome was the highest (13.79%). In particular, note that 62.16% of the patients' syndromes in group 3 always remained at the heat stage (Figure 5(c)).

### 3.4. Chinese Herb Medicine Characteristics of T2D Patients in Heat Stage

**Figure 6:**
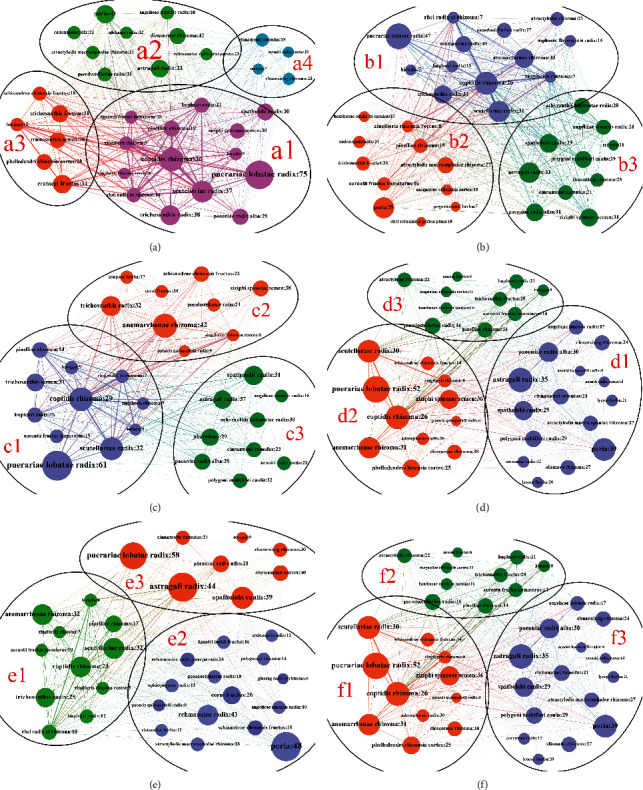
Chinese herb medicine complex network community division of T2D patients with different courses in the heat stage. For example, “Coptidis Rhizoma: 28.8”—the number after the node name in Figure 6 represents the drug dose. (a) Chinese herb medicine community division of patients with unchanged syndromes in group 1. (b) Chinese herb medicine community division of patients with syndromes change in group 1. (c) Chinese herb medicine community division of patients with unchanged syndromes in group 2. (d) Chinese herb medicine community division of patients with syndromes change in group 2. (e) Chinese herb medicine community division of patients with unchanged syndromes in group 3. (f) Chinese herb medicine community division of patients with syndromes change in group 3.

In the syndrome evolution Sanji diagram, our results show that more than 60% of the patients in the three groups have no change in syndrome, and other patients have syndrome transformation. In this case, we divided all patients' drugs of three groups into two categories: syndrome unchanged group (Figures 6(a), 6(c), and 6(e)) and syndrome change group (Figures 6(b), 6(d), and 6(f)).

The Chinese herb communities **a1**, **c1**, and **e1** are the largest communities, accounting for 42.42%, 37.93%, and 39.13%. All the drugs in communities **a1**, **c1**, and **e1** have the effect of clearing away heat and invigorating fluid, and Coptidis Rhizoma, Scutellariae Radix, Anemarrhenae Rhizoma, Trichosanthis Radix, and Puerariae Lobatae Radix have large node degree. Communities **a2** (27.27%), **c2** (31.03%), and **e2** (37.35%) all contain drugs with the effect of tonifying qi and nourishing yin. The difference is that community **e2** includes more drugs for nourishing kidney yin, such as Rehmanniae Radix, Corni Fructus, Polygonati Rhizoma, and Cistanches Herba. The drugs belonging to communities **a4** (12.12%), **c3** (31.03%), and **e3** (23.53%), such as Astragali Radix, Cinnamomi Ramulus, Spatholobi Caulis, and Chuanxiong Rhizoma, are all drugs with the effect of tonifying qi or promoting blood circulation. However, the proportion of community **a4** in patients with no syndromes change of group 1 was very small. Chinese herb community **a3** (18.18%) included *Trichosanthes*, Hawthorn, and *Monascus*, which mainly had the effect of resolving phlegm (Figures 6(a), 6(c), and 6(e)).

In group 1, the drugs included in Chinese herb communities **b2** (32.35%) and **a3** (18.18%) were phlegm-dissipating drugs, but the proportion of **b2** was higher than that of **a3** (Figures 6(a) and 6(b)). Blood-activating drugs are included in community **b3** (29.41%) and community **a4** (12.12%), but the proportion of **b3** is higher than that of **a4** (Figures 6(a) and 6(b)). In Figures 6(d) and 6(f), **d1** (41.67%) and **f1** (35.14%) are the largest Chinese herb communities, mainly including drugs for benefiting qi and activating blood, which are quite different from the results shown in Figures 6(c) and 6(e).

## 4. Discussion

In this study, 228 T2D patients were divided into three groups based on the course of disease: within 5 years, 5–10 years, and more than 10 years. Moreover, with the increase of the course of the disease, the age of the patients also increased, and the patients with a course of disease of more than 10 years were all over 40 years of age. According to the results in Section 3.2, regardless of the course of disease, the basic syndrome of T2D patients in the heat stage is liver-stomach heat syndrome. Moreover, heat often does not exist alone but is combined with phlegm, dampness, qi stagnation, and blood stasis. Therefore, according to the specific conditions of different patients, there will be different types of compound heat syndrome such as phlegm-heat, damp-heat, stagnant-heat, and stasis-heat syndrome. The studies in [20–23] have shown that there is obvious high expression of tumor necrosis factor-*α* (TNF-*α*), interleukin-6 (IL-6), C-reactive protein (CRP), and other inflammatory factor-related genes in TCM heat syndrome, and more and more studies [24–26] suggest that T2D may be an inflammatory response mediated by cytokines and is an immune disease. In particular, the proportion of overweight and obese patients included in this study is as high as 70%. Obesity can cause chronic low-grade inflammation in adipose tissue, liver, and pancreas, leading to insulin resistance and T2D [27, 28], and obese patients show symptoms of phlegm-dampness and heat in TCM [29]. Zhou et al.'s [30] prospective study on TCM syndromes of early T2D patients showed that obese patients were more common in early T2D, and phlegm-heat syndrome was the main syndrome type in early T2D. In addition, there were more male patients in this study. The studies in [31] have shown that the majority of male patients with T2D are dampness-heat constitutionally, so they are more prone to dampness-heat syndrome.

There was a significant relationship between the direction of syndrome evolution and the duration of the disease in patients with T2D in the heat stage. First of all, patients with a disease course of 5 years and 5–10 years are more likely to deteriorate to the syndrome of deficiency of spleen and stomach qi and Yin. Heat consumes qi and yin in TCM theory, which leads to the syndrome transformation from heat syndrome to qi deficiency and yin deficiency syndrome. This is clearly stated in a large number of ancient books of TCM and the views of modern Chinese medicine scholars [32–34]. Although the syndrome of patients with a course of disease of more than 10 years is also easy to transform to yin deficiency syndrome, its transformation direction is kidney yin deficiency syndrome. This is significantly different from those of the first two groups. TCM theory believes that the disease for a long time is easy to damage the kidney yin, the origin of the whole body yin fluid. For patients with a course of more than 10 years, the heat syndrome lasts so long that it damages the kidney yin, so the disease location of the yin deficiency syndrome has changed. In addition, the syndrome of patients greater than 10 years is also easily converted to the direction of qi deficiency. The occurrence of blood stasis is related to a variety of factors, such as heat injuring body fluid, phlegm-dampness blocking meridians, and impaired viscera function [35]. Moreover, with the gradual increase of the course of the disease, the meridian obstruction of the patients became serious incrementally, and finally blood stasis became the main factor leading to diabetic complications such as retina, nephropathy, coronary heart disease, and myocardial infarction [36]. The studies in [37] have shown that patients with blood stasis syndrome have more serious disorders of fat metabolism, amino acid metabolism, and energy metabolism than those without blood stasis syndrome, and blood stasis syndrome is common in T2D patients with a course of 10–15 years [38].

When discussing the medication characteristics of the patient, we discuss them separately according to whether the patient's syndrome has changed or not. In Section 3.2, more than 60% of the patients in all three groups had no change in syndrome after treatment, which means that the Chinese herb medicine used by these patients helps in keeping their condition stable. Here, we believe that patients whose syndromes do not deteriorate after taking Chinese herbs, to some extent, demonstrate the effectiveness of TCM or at least prove that TCM is beneficial in keeping their condition stable. For this chronic disease, portraying “cure of diabetes” as a goal for all persons with diabetes, however, is misleading and has the potential to do harm [39]. Even for insulin, it is not a cure for diabetes; it is just a treatment [40]. *Standards of Medical Care in Diabetes-2020* [41] mentioned the following: “The goals of treatment for diabetes are to prevent or delay complications and optimize quality of life.” There is no doubt that keeping the patient's condition stable helps to delay complications. Therefore, in our results, we found that all those patients were treated with antipyretic drugs, such as Coptidis Rhizoma, Scutellariae Radix, Anemarrhenae Rhizoma, Trichosanthis Radix, and Puerariae Lobatae Radix. The studies in [42] have shown that heat-clearing herbs can generally reduce the abundance and species diversity of intestinal flora, maintain intestinal barrier function, reduce inflammatory reaction, and improve insulin resistance by regulating intestinal flora disorder. For example, regarding Coptidis Rhizoma, one of the representative heat-clearing drugs, its core component berberine in reducing blood lipids and improving insulin resistance has been fully proved in many randomized clinical trials [43]. In addition, studies have proved that the therapeutic effect of Coptidis Rhizoma is closely related to the dose, and the significant hypoglycemic effect can be observed when the dose is high [44]. In our results, the average high dose of *Coptis chinensis* in heat-stage T2D patients was 24–28 g. However, heat-clearing herbs are cold, especially for T2D patients with weak function of spleen and stomach, which should not be taken for a long time. Therefore, a commonly used compatibility is a combination of heat-clearing herbs and warm herbs such as Zingiberis Rhizoma and Pinelliae Rhizoma to neutralize the cold nature of heat-clearing herbs. The studies in [45] have shown that this compatibility can increase the expression of insulin receptor in the pancreas of T2D model mice and improve insulin resistance. Secondly, patients in the hot stage tend to evolve in the direction of qi and yin deficiency syndrome. Therefore, the three groups of patients also used Pseudostellariae Radix, Astragali Radix, Panacis Quinquefolii Radix, Glehniae Radix, and Ophiopogonis Radix, which have the effect of tonifying qi and nourishing yin. These Chinese herbs are compatible with heat-clearing drugs, the basic medication for T2D patients in the heat stage, which embodies the idea of “treating predisease” in TCM. However, for patients with a disease course of more than 10 years, additional traditional Chinese medicines with kidney yin nourishing effect should be added to cope with their possible deterioration to kidney yin deficiency syndrome at any time, such as Corni Fructus, Schisandrae Chinensis Fructus, Ligustri Lucidi Fructus, Polygonati Rhizoma, and Angelicae Sinensis Radix.

With the exception of 60% of the patients who are in a more stable condition, the rest of the patients inevitably have syndrome changes even after treatment, which means that the disease has taken a turn for the worse. We compared the drugs used in two parts of patients with different conditions and found that the proportion of blood-activating drugs used in patients with worsening conditions increased significantly. In group 2 and group 3, for example, the blood-activating drugs became the most commonly used drugs rather than the generally thought heat-clearing drugs. Therefore, it is valid to consider that blood stasis may be the main factor leading to the disease deterioration in patients with T2D in the heat stage. Consequently, in order to prevent the gradual accumulation of blood stasis leading to aggravation of the condition, many Chinese medical experts [46–48] believe that patients should use drugs to promote blood circulation and remove blood stasis as soon as possible. In our study, except for heat-clearing drugs as basic drugs, patients with more stable conditions used drugs for promoting blood circulation and removing blood stasis, and the longer the course of disease, the greater the proportion of use. The representative drugs are Astragali Radix, Cinnamomi Ramulus, Spatholobi Caulis, Chuanxiong Rhizoma, *Hirudo*, and *Pheretima*. The studies in [49] have shown that “Buqi Huoxue recipe” can improve glucose and lipid metabolism, reduce the level of oxidative stress in myocardial tissue to a certain extent, downregulate the expression of some inflammatory factors in blood, and improve the related symptoms of diabetic angiopathy in many aspects.

In addition, we also found an interesting result that high-dose Puerariae Lobatae Radix is often used to treat T2D, with an average of 41–60 g. Puerariae Lobatae Radix not only is nontoxic but also has the dual effects of clearing heat and nourishing yin. Puerarin, the core component of Puerariae Lobatae Radix, may directly benefit DM by decreasing blood glucose levels, improving insulin resistance, protecting islets, inhibiting inflammation, decreasing oxidative stress, and inhibiting Maillard reaction and advanced glycation end products (AGEs) formation [50]. But its efficacy is low, so it needs to be used in large doses or in combination with other herbs. For example, Gegen Qinlian Decoction, composed of Coptidis Rhizoma, Scutellariae Radix, Puerariae Lobatae Radix, and Glycyrrhizae Radix et Rhizome, is a classic traditional Chinese medicine prescription that is widely used to clinically treat diabetes mellitus, and it has been proved that it can significantly reduce blood glucose and increase serum insulin level in type 2 diabetic mice [51].

## 5. Conclusion

The basic syndrome of T2D patients in the heat stage is liver-stomach heat syndrome, and heat is often combined with phlegm, dampness, qi stagnation, and blood stasis. Therefore, patients generally have phlegm-heat, dampness-heat, stagnation-heat, and stasis-heat syndrome as well as other compound-heat syndromes. In the process of syndrome evolution, the patients mainly evolved to the syndrome of qi-yin deficiency. But, for patients with a course of more than 10 years, because of their longer course of disease, they tend to evolve in the direction of deficiency of qi and blood stasis or syndrome of liver-kidney yin deficiency. Drugs that can help T2D patients in the heat stage to maintain their condition stable are heat-clearing drugs represented by *Coptis chinensis*, which usually need to be combined with warming interior drugs such as Zingiberis Rhizoma and Pinelliae Rhizoma. At the same time, according to the different syndrome evolution trends of patients with different courses, patients with shorter course of disease should appropriately increase the proportion of herbs for tonifying qi and nourishing yin, while patients with longer course of disease can use more herbs for nourishing kidney yin. In addition, we also found that blood stasis was the main factor leading to the deterioration of the condition of T2D patients in the heat stage. Therefore, for patients in the heat stage, it is also necessary to use blood-activating drugs to prevent the deterioration of the disease, and representative drugs include Astragali Radix, Spatholobi Caulis, Cinnamomi Ramulus, Chuanxiong Rhizoma, and *Pheretima*.

The advantage of this study is that we introduce a new data mining method, Sangji diagram, to study the evolution of TCM syndromes. Moreover, we have made a complete study of the syndromes distribution, syndromes evolution, and the corresponding herb characteristics and doses in the heat stage of T2D, which is more helpful for doctors to use in clinical treatment according to the actual situation of patients. However, an obvious disadvantage of our research is that we have a small amount of data. This is mainly due to our strict data filtering conditions. Moreover, we did not control for some variables that might affect the results. For example, we think that the drugs of patients with unaltered symptoms will help them stabilize their condition, but in fact regular diet and exercise may also improve their condition.

## Figures and Tables

**Table 1:** Basic information of patients.

Basic information	Classification	Group 1	Group 2	Group 3	*χ* ^2^ value	*P* value
Gender	Male	76 (33.33%)	49 (21.49%)	23 (10.09%)	0.293	0.864
	Female	42 (18.42%)	24 (10.53%)	14 (6.14%)		

Age	(18∼40)	38 (16.67%)	6 (2.63%)	0 (0%)	40.769	<0.001
	(40∼60)	57 (25%)	42 (18.42%)	14 (6.14%)		
	≥60	23 (10.09%)	25 (10.96%)	23 (10.09%)		

BMI	18.5∼23.9	26 (16.99%)	15 (9.8%)	3 (1.96%)	4.174	0.383
	24.0∼27.9	36 (23.53%)	19 (12.42%)	13 (8.5%)		
	≥28	19 (12.42%)	15 (9.8%)	7 (4.58%)		

BMI: body mass index. The classification criteria come from *the Guidelines for the Prevention and Control of Overweight and Obesity in Chinese Adults* [19]: BMI = 24 is the limit of overweight for Chinese adults, and BMI ≥ 28 is the limit of obesity. BMI records of 75 patients were missing in this study.

## Data Availability

The data used to support the findings of this study are included within the article.
